# Cerebrospinal fluid metabolomics identifies a key role of isocitrate dehydrogenase in bipolar disorder: evidence in support of mitochondrial dysfunction hypothesis

**DOI:** 10.1038/mp.2015.217

**Published:** 2016-01-19

**Authors:** N Yoshimi, T Futamura, S E Bergen, Y Iwayama, T Ishima, C Sellgren, C J Ekman, J Jakobsson, E Pålsson, K Kakumoto, Y Ohgi, T Yoshikawa, M Landén, K Hashimoto

**Affiliations:** 1 Division of Clinical Neuroscience, Chiba University Center for Forensic Mental Health, Chiba, Japan; 2 Qs' Research Institute, Otsuka Pharmaceutical, Tokushima, Japan; 3 Department of Medical Epidemiology and Biostatistics, Karolinska Institutet, Stockholm, Sweden; 4 Laboratory for Molecular Psychiatry, RIKEN Brain Science Institute, Wako, Japan; 5 Department of Physiology and Pharmacology, Karolinska Institutet, Stockholm, Sweden; 6 Department of Clinical Neuroscience, Karolinska Institutet, Stockholm, Sweden; 7 Institute of Neuroscience and Physiology, Section of Psychiatry and Neurochemistry, University of Gothenburg, Gothenburg, Sweden; 8 Tokushima Research Institute, Otsuka Pharmaceutical, Tokushima, Japan

## Abstract

Although evidence for mitochondrial dysfunction in the pathogenesis of bipolar disorder (BD) has been reported, the precise biological basis remains unknown, hampering the search for novel biomarkers. In this study, we performed metabolomics of cerebrospinal fluid (CSF) from male BD patients (*n*=54) and age-matched male healthy controls (*n*=40). Subsequently, post-mortem brain analyses, genetic analyses, metabolomics of CSF samples from rats treated with lithium or valproic acid were also performed. After multivariate logistic regression, isocitric acid (isocitrate) levels were significantly higher in the CSF from BD patients than healthy controls. Furthermore, gene expression of two subtypes (*IDH3A* and *IDH3B*) of isocitrate dehydrogenase (IDH) in the dorsolateral prefrontal cortex from BD patients was significantly lower than that of controls, although the expression of other genes including, aconitase (*ACO1*, *ACO2*), *IDH1*, *IDH2* and *IDH3G*, were not altered. Moreover, protein expression of IDH3A in the cerebellum from BD patients was higher than that of controls. Genetic analyses showed that *IDH* genes (*IDH1*, *IDH2*, *IDH3A*, *IDH3B*) and *ACO* genes (*ACO1*, *ACO2*) were not associated with BD. Chronic (4 weeks) treatment with lithium or valproic acid in rats did not alter CSF levels of isocitrate, and mRNA levels of *Idh3a*, *Idh3b*, *Aco1* and *Aco2* genes in the rat brain. These findings suggest that abnormality in the metabolism of isocitrate by IDH3A in the mitochondria plays a key role in the pathogenesis of BD, supporting the mitochondrial dysfunction hypothesis of BD. Therefore, IDH3 in the citric acid cycle could potentially be a novel therapeutic target for BD.

## Introduction

Bipolar disorder (BD) is a major psychiatric disease characterized by episodes of depression and mania or hypomania interspaced by periods of euthymia. With a typical age of onset in late adolescence or early adulthood, BD is a major health problem that requires continuous monitoring and often lifelong treatment,^1, 2, 3^ and places a substantial economic burden on health-care systems and society.^4^ A recent Swedish resource use study estimated the average annual cost to 28 011 euro/year/patients^5^ and a 2009 US study estimated the direct and indirect costs of BD to be 151 billion dollars.^6^

Although a number of studies of families and twins show the importance of genetic factors affecting susceptibility to BD, the precise pathogenesis of BD is not well understood.^7^ BD may be a neuroinflammatory disorder^8^ in which relapses are toxic, indicating the importance of early detection to prevent an otherwise negative prognosis.^9^ Accumulating evidence suggests that mitochondrial dysfunction plays a key role in the pathogenesis of BD.^10, 11, 12, 13, 14, 15^ A number of findings (for example, calcium dysregulation, disturbed energy metabolism, oxidative phosphorylation abnormalities and abnormalities in cellular resilience and synaptic plasticity) from microarray studies, biochemical studies, neuroimaging studies and post-mortem brain studies all support the role of mitochondrial dysfunction in the pathogenesis of BD.^10, 11, 12, 13, 14, 15^

Metabolomics is the profiling of small-molecule metabolites and provides the potential to characterize specific metabolic phenotypes associated with a disease. Metabolomics has an advantage over other ‘omics' techniques in that it directly samples the metabolic changes in an organism and integrates information from changes at the gene, transcript and protein levels, as well as posttranslational modifications.^16, 17, 18^ Lan *et al.*^19^ reported metabolomics analysis of post-mortem brain samples from BD patients and controls. In this study, they found that levels of *myo*-inositol, creatine, glutamate, lactate and phosphocholine were increased in the post-mortem brain from BD patients (*n*=10), suggesting mitochondrial dysfunction in BD.^19^ Tissue concentration of small molecules, such as amino acids, in brain samples is known to be significantly affected by post-mortem interval as the metabolic rate of these small molecules is fast.^20, 21^ For example, levels of L-serine and glycine in the brain increase with increasing length of post-mortem interval, whereas levels of D-serine and glutamate decrease with increasing length of post-mortem interval.^21^ Therefore, metabolomic analyses using post-mortem brain samples are not useful for determination of biomarkers. Cerebrospinal fluid (CSF) is arguably the most relevant sampling substrate for the *in vivo* study of brain disorders as it reflects the metabolic status and the biochemistry of the brain. Metabolomic profiles of CSF in patients and controls therefore have the potential to reveal protein differences linked to the pathogenesis of BD that might have value as biomarkers.

Capillary electrophoresis time-of-flight mass spectrometry (CE-TOFMS) is a state-of-the-art metabolome analysis.^22^ The advantages of CE-TOFMS analysis include extremely high resolution, versatility and ability to simultaneously quantify virtually all the charged low-molecular-weight compounds in a sample.^23^ Two studies using this technique showed robust changes in four molecules (arginine, taurine, 5-oxoproline and lactic acid) in the plasma of autism spectrum disorders,^24^ and significant changes in the five molecules (creatine, betaine, nonanoic acid, benzoic acid and perillic acid) in the plasma of first-episode, medicated patients with schizophrenia.^25^ However, there are no reports using this technique in CSF samples from BD patients.

In the present study, we performed metabolomics assays using CE-TOFMS of CSF samples of mood-stabilized BD patients and age-matched healthy controls. Furthermore, we performed the gene and protein expression analyses in post-mortem brain samples and genetic association analyses of the genes relevant to the substance identified by CSF metabolomics. In order to examine the effect of medication on metabolites, we also performed metabolomics characterization of CSF samples from rats chronically treated with lithium (Li) or valproic acid (VPA).

## Materials and methods

### Participants

The BD patients were recruited from the St Göran Bipolar Project, enrolling patients from the bipolar unit at the Northern Stockholm Psychiatric Clinic, Stockholm, Sweden. The work-up and diagnostic assessments have been described in detail previously.^8, 26, 27^ The key clinical assessment instrument used was the ADE (Affective Disorder Evaluation), developed for the STEP-BD (Systematic Treatment Enhancement Program of Bipolar Disorder).^28^ The full diagnostic assessment was based on all available sources of information including patient interview, case records and, if possible, interviews with the next of kin. To reduce inter-rater bias, a best-estimate diagnostic decision based on all information available at admission was made at a diagnostic case conference by a consensus panel of experienced board-certified psychiatrists (*n*=2–5) specialized in BD.

The general criteria for inclusion were: (1) age of at least 18 years and (2) meeting the Diagnostic and Statistical Manual (DSM)-IV criteria for bipolar spectrum disorder (that is, type I, type II and not otherwise specified). Information regarding age, sex, number of lifetime manic/hypomanic/depressive/total episodes, duration of illness (defined as years since first hypomanic or manic episode), body mass index and previous psychotic episodes was collected. The severity of BD was rated using the CGI (Clinical Global Impression) rating scales and GAF (Global Assessment of Functioning). For ethical reasons, patients continued to take their prescribed medications at the time of CSF sampling.

Population-based controls were randomly selected by SCB (Statistics Sweden) and contacted by mail. Given an expected response rate of 1:7, seven invitations were sent out per enrolled subject. Of the invited controls, 14% responded to the invitation and were subjected to a preliminary telephone screening by a research nurse to exclude severe mental health conditions, neurological diseases, and substance abuse. Eligible individuals were scheduled for a 1-day comprehensive assessment where they underwent a psychiatric interview by experienced clinicians using the MINI (Mini-International Neuropsychiatric Interview) to exclude psychiatric disorders.^29^ Substance abuse was screened for at the telephone interview by the nurse, in the psychiatric interview, by the AUDIT (Alcohol Use Disorders Identification Test) and the DUDIT (Drug Use Disorders Identification Test), as well as by determining serum levels of carbohydrate-deficient transferrin.^30^ Overconsumption of alcohol as revealed by carbohydrate-deficient transferrin or responses indicating large consumption (>8 standard drinks per time more than 2 times per week), and/or amnesia and/or loss of control more than once per month resulted in the exclusion of these individuals from the study. Other exclusion criteria were neurological conditions other than mild migraines, untreated endocrinological disorders, pregnancy, dementia, recurrent depressive disorder and suspected severe personality disorders (based on interview and the Structured Clinical Interview for DSM (SCID-II) screen personality assessment) and a family history of schizophrenia or BD in first-degree relatives.

The study was approved by the Regional Ethics Committee in Stockholm and conducted in accordance with the latest Helsinki Protocol. All patients and controls consented orally and in writing to participate in the study. Informed consent was obtained during a euthymic period (that is, during a time period when patients did not meet criteria for a depressive or manic episode). All patients were capable of freely giving fully informed consent, as determined by the physicians who enrolled the patients. A total of 54 male BD patients and 40 male healthy controls were included (Table 1).

### CSF sampling

CSF sampling (lumbar puncture) was performed when the participants were euthymic. Sampling occurred between 0900 and 1000 h after an overnight fast. To collect CSF, the spinal needle was inserted into the L3/L4 or L4/L5 interspace and a standardized volume of 12 ml CSF was collected in a polypropylene tube, gently inverted to avoid gradient effects and divided into 1.0–1.6 ml aliquots in polypropylene tubes. The aliquoted CSF samples were stored at −80 °C pending analysis at the Biobank at Karolinska Institute, Stockholm, Sweden. An identical procedure was performed for the controls. The samples were stored at −80 °C until delivered by courier mail, frozen on dry ice, to Chiba University, Japan, for metabolomics analysis. This study was approved by research ethics committee of the Graduate School of Medicine, Chiba University.

### Metabolomic profiling of human CSF samples

Metabolomic analyses of CSF samples from healthy controls and BD patients were performed using the CE-TOFMS at Human Metabolome Technologies (Yamagata, Japan). In this study, 116 major metabolic compounds from various pathways (glycolytic system, pentose phosphate pathway, citric acid cycle, urea cycle, polyamine–creatine metabolism pathway, purine metabolism pathway, glutathione metabolism pathway, nicotinamide metabolism pathway, choline metabolism pathway and diverse amino acid metabolism pathway) were selected for metabolomics analysis (Supplementary Tables S1 and S2). Detailed methods are shown in the Supplementary Methods.

### Expression of *IDH* and *ACO* genes in the dorsolateral prefrontal cortex

Post-mortem brain samples from Brodmann's area (BA) 46 were obtained from the Stanley Medical Research Institute (http://sncid.stanleyresearch.org/).^31, 32^ Brain samples were taken from BD patients (*n*=35) and controls (*n*=34) (Supplementary Table S3). Details are shown in the Supplementary Methods.

### Protein expression of IDH3A and IDH3B in the post-mortem brain samples

Cerebellum and parietal cortex (BA7) from BD (*n*=15), major depressive disorder (*n*=15), schizophrenia (*n*=15) and normal controls (*n*=15)(Supplementary Table S4) were obtained from the Stanley Medical Research Institute (http://sncid.stanleyresearch.org/).^31, 32, 33^ Details are shown in the Supplementary Methods.

### Genetic association analyses of *IDH* and *ACO* genes in BD patients and controls

Details are shown in the Supplementary Methods.

### Metabolomics of rat CSF samples and expression of genes for *Idh* and *Aco* in rat brain samples

Details are shown in the Supplementary Methods.

### Statistical analyses

Data from human samples are presented as mean±s.d. Statistical analysis was performed using SAS software version 9.3 (SAS Institute, Cary, NC, USA). Analyses of metabolites between control and BD groups were performed using unpaired *t*-tests (Supplementary Table S2) and Wilcoxon rank-sum tests. A logistic regression model with a stepwise selection method was used for the multivariate analysis.^34, 35^ In consideration, we checked it about basic assumptions for multivariate logistic regression model include independence of errors, absence of multicollinearity and lack of strongly influential outliers. The adequacy of the fitted model was performed by goodness-of-fit tests used the Hosmer–Lemeshow test for binary response data in which a *P*-value >0.1 indicates a good fit.^36^ Before reaching definitive conclusions from the results of this method, the jack-knife method (a performance evaluation method in which a measured value predicted from the *n*-1 observations, removing the own predicted observation: leave-one-out cross validation) was used to quantify the model's internal validity.^37^ Finally, results for independent variables were typically reported as odds ratios with 95% confidence intervals. To investigate whether the parameter (isocitrate) selected by logistic regression is affected by clinical data or medication in BD patients, additional multiple regression analyses were performed. The variables are as indicated below: the age at first symptoms, GAF score, total number of episodes (depressive, hypomanic, manic and mixed), MADRS (Montgomery–Åsberg Depression Rating Scale) score, YMRS (Young Mania Rating Scale) score, % of psychotic episodes and medication (mood stabilizer, Li, anticonvulsant, VPA, lamotorigine, antidepressant, anxiolytic and antipsychotic).

The unpaired *t*-test was used to evaluate changes in expression levels of the *ACO* and *IDH* genes between control and BD groups. False discovery rate was used to control for multiple comparisons as indicated in the results^38^ and *P<*0.05 was considered statistically significant. Rat data are also presented as mean±s.d. To determine the effects of drug treatment, a one-way analysis of variance or analysis of covariance followed by the *post hoc* Dunnett's test was used. *P-*values of <0.05 for two-tailed tests were considered statistically significant.

## Results

### Metabolomics of CSF samples from BD patients and controls

**Figure 1:**
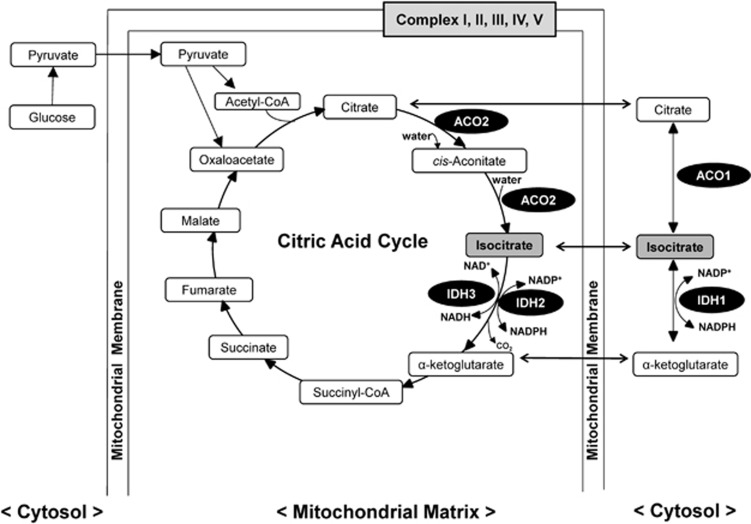
Metabolic pathway of citrate and isocitrate on the citric acid cycle in the mitochondrial matrix and in the cytosol. ACO1 and ACO2 are localized in the cytosol and the mitochondrial matrix, respectively. ACO1 interconverts citrate and isocitrate in the cytosol, allowing the cell to balance the amount of NADPH generated from isocitrate by IDH1. ACO2 is an enzyme that catalyzes citrate to isocitrate via *cis*-aconitate in the citric acid cycle. IDH1 is localized in the cytosol, and IDH2 and IDH3 are found in the mitochondrial matrix. The IDH1 and IDH2 enzymes catalyze a redox reaction that converts isocitrate to α-ketoglutarate (also known as 2-oxoglutarate (2-OG)), while reducing NADP^+^ to NADPH and liberating CO_2_. The mitochondrial IDH3 enzyme is an essential element of the citric acid cycle, catalyzing the oxidation of isocitrate to α-ketoglutarate with the reduction of NAD^+^ to NADH. The electron transfer chain (ETC) in the mitochondrial membrane is a series of complexes, I–V, that transfer electrons from electron donors to electron acceptors via redox reactions.

First, we performed metabolomics analyses of CSF samples from 40 healthy controls and 54 BD patients. There were no differences between healthy controls and BD patients for age and body mass index (Table 1). Table 1 shows demographics and clinical characteristics of the BD patients. We measured 116 major metabolic substances in various pathways, of which 72 were detected in CSF, and the remaining 44 were under the detection limit (Supplementary Tables S1 and S2). To select the substances showing significant differences between healthy controls and BD patients, we first performed both unpaired *t*-tests and Wilcoxon rank-sum tests between healthy controls and BD patients. Thirteen compounds, including uric acid, fructose 6-phosphate, ribose 5-phosphate, CoA, 2-oxoisovaleric acid, lactic acid (lactate), pyruvic acid (pyruvate), citric acid (citrate), isocitric acid (isocitrate), *cis*-aconitic acid (*cis*-aconitate), urea, alanine and tryptophan, were significantly altered (Supplementary Table S2). Pyruvate, citrate, isocitrate and *cis*-aconitate are molecules in the citric acid cycle (also known as the Krebs cycle) (Figure 1).

Multivariate logistic regression analysis was performed to evaluate the association between the 13 metabolites and BD (Supplementary Table S5). A stepwise selection-elimination method was used, and the significance level was set at 5%. One parameter, isocitrate, was independently associated with BD (Table 2, the resulting equation is shown in the footnote). In this model, the Hosmer–Lemeshow goodness-of-fit statistic (the adequacy of the fitted model) was 12.145 with 8 degrees of freedom (*P*=0.145), indicating a good fit of the model. The increased CSF level of isocitrate was significantly correlated with an increased risk of BD (odds ratio, 4.402; 95% confidence interval, 2.249–7.266).

To investigate whether isocitrate is affected by clinical data or medication in BD patients, we performed additional multiple regression analyses. The variables are as indicated below: the age at first symptoms, GAF score, total number of mood episodes (depressive, hypomanic, manic and mixed), MADRS score, YMRS score, AUDIT total score, DUDIT total score, psychotic episodes, family history (for example, bipolar and unipolar), alcohol dependence, alcohol use, substance abuse and medication (mood stabilizer, Li, anticonvulsant, VPA, lamotrigine, antidepressant, anxiolytic and antipsychotic). Consequently, any variables were not selected (Supplementary Table S6). Furthermore, there was also no correlation between plasma lithium concentration and CSF isocitrate levels in BD patients. As isocitrate was significantly (*P<*0.0001) altered in the CSF from BD patients after logistic regression, we focused on isocitrate for subsequent analyses.

### Expression of *ACO* and *IDH* genes in the dorsolateral prefrontal cortex of BD patients and controls

Isocitrate is synthesized from citrate via *cis*-aconitate by the enzyme aconitase (ACO: aconitate hydratase) (Figure 1). Two isozymes of aconitase are present in mammalian cells: the mitochondrial enzyme (m-aconitase: ACO2) that functions in the citric acid cycle, and the bifunctional cytosolic enzyme (c-aconitase/IRP1: ACO1) that also plays a role in the regulation of iron metabolism.^39^ Isocitrate dehydrogenase (IDH) catalyzes the oxidative decarboxylation of isocitrate, producing α-ketoglutarate (also known as 2-oxoglutarate) and CO_2_ (Figure 1). In humans, IDH exists in three forms. The two isoforms, which are mutated in cancer, IDH1 and IDH2, utilize this catalytic process in additional contexts including metabolism and glucose sensing (IDH1) and regulation of oxidative respiration (IDH2). IDH3 primary functions as the allosterically regulated, rate-limiting enzymatic step in the citric acid cycle, while converting NAD^+^ to NADH in the mitochondria. IDH3 is a heterotetramer with two α-subunits (IDH3A), one β-subunit (IDH3B) and one γ-subunit (IDH3G)^40^ (Figure 1).

In this study, we measured mRNA levels of *ACO* and *IDH* genes in the dorsolateral prefrontal cortex from BD patients (*n*=35) and controls (*n*=34). The mRNA levels of *ACO1* (*P*=0.178) and *ACO2* (*P*=0.487) in the dorsolateral prefrontal cortex from BD patients were not different from those of controls. The mRNA levels of *IDH3A* (*P*=0.007) and *IDH3B* (*P*=0.007) in the BD group were significantly lower than that of control group (Table 3A and B). Furthermore, analysis of covariance (adjusted for pH and post-mortem interval) showed that the mRNA levels of *IDH3A* and *IDH3B* were significantly lower in the BD group compared with the control group. In contrast, mRNA levels of *IDH3G* (*P*=0.422), *IDH1* (*P*=0.755), and *IDH2* (*P*=0.279) were not different between the two groups (Table 3A).

### Protein expression of IDH3A and IDH3B in the cerebellum and parietal cortex of BD patients and controls

We examined whether protein expression of IDH3A and IDH3B in the cerebellum and parietal cortex (BA7) differed between samples from patients with BD, major depressive disorder or schizophrenia, and controls (Supplementary Table S4). Results showed that protein levels of IDH3A in the cerebellum from BD (*P*=0.017), major depressive disorder (*P*=0.006) and schizophrenia (*P*=0.001) were significantly lower than in controls (Table 3B). In contrast, one-way analysis of variance showed no significant changes on the protein level of IDH3B in the cerebellum. There were also no differences for IDH3A and IDH3B levels in the parietal cortex among the four groups (Table 3B).

### Genetic analyses of *ACO* and *IDH* genes in BD and controls

We performed genetic association analyses of *ACO* and *IDH* genes in BD patients (sample set 1: *n*=1415, sample set 2: *n*=836) and controls (sample set 1: *n*=1271, sample set 2: *n*=2093). The call rates for all markers were >98%, and none showed marked departures from Hardy–Weinberg equilibrium. Across all single-nucleotide polymorphisms tested, several single-nucleotide polymorphisms within the *ACO1* and *IDH2* genes attained nominal significance, and none remained significant following multiple testing correction (Supplementary Table S7).

Subsequently, epistasis tests were conducted separately in the two sample sets. It is worth noting that the compelling interactions in both sample sets were detected between markers in the *ACO1* and *IDH2* genes (sample set 1: *P*=0.007 for rs10970986 and rs2970357; sample set 2: *P*=0.003 for rs13302577 and rs2970359) (Supplementary Table S8).

### Effects of Li and VPA on rat CSF levels, and expression of *Aco1*, *Aco2*, *Idh3a* and *Idh3b* genes in the rat brain

In order to examine the effects of medication on the metabolites such as isocitrate, we performed metabolomics analyses of CSF samples from rats treated with chronic (4 weeks) treatment of Li or VPA. We measured the CSF concentrations of 116 major metabolites from several pathways. Sixty-four substances were detected in the rat CSF. Treatment with Li significantly increased CSF levels of succinic acid and argininosuccinic acid. On the other hand, treatment with VPA significantly altered CSF levels of threonine, glutamine, arginine, tryptophan and argininosuccinic acid (Supplementary Table S9). Thus, CSF levels of isocitrate in rats were not altered by chronic treatment with Li or VPA (Supplementary Table S9).

Next, we examined the effect of Li and VPA treatment on mRNA levels of *Aco1*, *Aco2*, *Idh3a* and *Idh3b* in the rat brain. The mRNA levels of *Aco1*, *Aco2*, *Idh3a* and *Idh3b* in the prefrontal cortex and hippocampus were not altered by treatment of Li or VPA (Supplementary Table S10). These results show that chronic treatment of Li and VPA did not affect the expression of mRNA for *Aco1*, *Aco2*, *Idh3a* and *Idh3b* in the prefrontal cortex and hippocampus of rats.

## Discussion

The major finding of this study was that CSF levels of isocitrate in BD patients were significantly higher than those in healthy controls, which was unrelated to medication. In addition, mRNA levels of *IDH3A* and *IDH3B* genes in the dorsolateral prefrontal cortex from BD patients were significantly lower than those of control samples. Furthermore, protein levels of IDH3A in the cerebellar tissue from BD patients were lower than those in controls. Altered isocitrate metabolism does not appear to result from primary genetic variations as single-nucleotide polymorphisms in the *ACO* and *IDH* genes were not associated with BD in the Swedish population. Furthermore, we found that chronic (4 weeks) treatment of Li or VPA did not alter CSF levels of isocitrate in the rats or the expression of mRNA of *Aco1*, *Aco2*, *Idh3a* and *Idh3b* in the prefrontal cortex and hippocampus of rat brain, suggesting that Li and VPA do not affect the synthesis and metabolism of isocitrate in the brain. Therefore, it is unlikely that these mood stabilizers affect CSF levels of isocitrate in BD patients. To our knowledge, this is the first study showing increased CSF levels of isocitrate in BD patients. We conclude that isocitrate could be a trait CSF biomarker for BD.

The IDH3 enzyme, localized in the mitochondria, plays a central role in the regulation of the citric acid cycle to produce the NADH required for oxidative phosphorylation (Figure 1).^41^ The mechanisms by which decreased expression of *IDH3A* and *IDH3B* affect the pathogenesis of BD remain largely unclear. In the citric acid cycle, NAD^+^-dependent IDH3 catalyzes the conversion of isocitrate to α-ketoglutarate, an essential reaction of the cycle that simultaneously changes NAD^+^ to NADH. The NADH produced in this step and other steps of the citric acid cycle is used to generate adenosine triphosphate (ATP), a molecule universally used in cells as an energy source.^42^ Mitochondrial oxidative phosphorylation is also the major ATP-producing pathway that supplies >95% of the total energy requirement in the cells. In this study, we did not find any changes of CSF levels of ATP, NAD^+^ and NADH in BD patients because they fell under the detection limit (Supplementary Table S2). A recent study using ^31^P-magnetic resonance spectroscopy showed that the effect of age on ATP concentration in the gray matter is significantly different between BD patients and healthy controls.^43^ Interestingly, we found decreased mRNA levels of *IDH3A* and *IDH3B* in the dorsolateral prefrontal cortex of BD, although we did not examine whether enzymatic activity of IDH3A and IDH3B is altered in the BD patients. We did find reduced expression of IDH3A protein in the cerebellum in BD and other psychiatric disorders such as major depressive disorder and schizophrenia. Thus, it is likely that IDH3A abnormalities may be involved in the pathogenesis of major psychiatric disorders. However, differences for IDH3B protein in the cerebellum and IDH3A in the parietal cortex did not reach statistical significance. Taken all together, it is likely that decreased activity of IDH3A in the brain may play a crucial role in the pathogenesis of BD, although further research on the role of IDH3 in BD is needed. Based on the central role of IDH3 in the citric acid cycle in the mitochondrial matrix (Figure 1), our findings support an abnormality in the mitochondrial function in BD. Thus, it seems that CSF isocitrate may index brain mitochondrial function in BD.

ATP is produced from oxygen and glucose through the citric acid cycle. The electron transfer chain (for example, complexes I–V) is the machinery by which mitochondria make ATP (Figure 1). The expression of mRNA-encoding mitochondrial proteins of electron transfer chain complexes I, III, IV and V was decreased in the post-mortem brain of BD patients as compared with controls.^44, 45^ Interestingly, decreased levels of electron transfer chain complex I and complex I activity in the prefrontal cortex from BD patients were also reported.^46^ These findings also suggest that mitochondrial dysfunction may be involved in the pathogenesis of BD.

Previous reports using ^31^P-magnetic resonance spectroscopy showed decreased levels of phosphocreatine^47^ and creatine,^48^ and increased levels of lactate^49^ in the brain of BD patients. However, we could not detect any changes in CSF levels of these substances after multivariate logistic regression. The reasons underlying these discrepancies may relate to differences in the techniques (CSF metabolomics vs ^31^P-magnetic resonance spectroscopy) or the samples (CSF vs specific brain regions).

Finally, there are some limitations to this study that need to be mentioned. First, only male subjects were enrolled in this study. It remains to be examined whether CSF levels of isocitrate are also altered in female BD patients. Second, it cannot be excluded that long-term medication with mood stabilizers, antidepressants and antipsychotics may affect CSF levels of metabolites, even though a 4-week treatment with Li or VPA did not alter CSF levels of isocitrate in rats. Therefore, further studies using a larger sample size of medication-free patients will be needed. Finally, we did not find any significant associations between the *IDH* (or *ACO*) genes and BD in a Swedish cohort of 2251 bipolar disorder patients and 3364 controls. It is possible, however, that there might be an association in other populations.

## Conclusions

Our study provides evidence for abnormality in the metabolism of isocitrate by IDH3A in the pathogenesis of BD. Therefore, mitochondrial IDH3 in the citric acid cycle may be a novel therapeutic target for BD.

## Figures and Tables

**Table 1:** Characteristics of the participants

	*Controls*		*Bipolar disorder (BD)*	
Number (male)	40^a^		54	
	*Median*	*IQR*	*Median*	*IQR*
Age (years)	36	30–47	41	32–52
BMI	24.1	22.8–25.8	25.7	24.1–28.1
*Diagnosis*			N	*%*
Bipolar disorder type I (BD I)			31	57.4
Bipolar disorder type II (BD II)			17	31.5
Not otherwise specified (NOS)			6	11.1
				
*Clinical data*			*Median*	*IQR*
Age of first symptoms			20	14–25
Depressive episodes^b^			6	3–10
Hypomanic episodes^c^			2	0–5
Manic episodes^c^			1	0–2
Mixed episodes^c^			0	0–25
GAF^c^			70	60–80
MADRS^d^			4	0–11
YMRS^e^			1	0–2
No. of episodes^c^			18	1–80
Audit total score^f^			8	2–11
Dudit total score^g^			0	0–0
			*N*	*%*
Psychosis episodes^c^			25	46.3
Family history of bipolar^a^			26	49.1
Family history of unipolar^a^			30	56.6
Alcohol dependence^b^			14	26.9
Alcohol abuse^b^			13	25.0
Substance abuse^b^			8	15.4
				
*Medication*			*N*	*%*
Mood stabilizer			44	81.5
Lithium (Li)			34	63.0
Anticonvulsants			19	35.2
Valproate (VPA)			7	13.0
Lamotrigine			12	22.2
Antidepressants			20	37.0
Anxiolytics			11	20.4
Antipsychotics			16	29.6

Abbreviations: BMI, body mass index; GAF, Global Assessment of Functioning; IQR, interquartile range; MADRS, Montgomery–Åsberg Depression Rating Scale; YMRS, Young Mania Rating Scale.

a Missing data for 1 individual in the control group.

b Missing data for 2 individuals in the patient group.

c Missing data for 1 individual in the patient group.

d Missing data for 10 individuals in the patient group.

e Missing data for 11 individuals in the patient group.

f Missing data for 9 individuals in the patient group.

g Missing data for 6 individuals in the patient group.

**Table 2:** Independent predictor in CSF samples of BD patients by logistic regression

*Parameter*	*Odds ratio (95% CI)*	P-*value*
Isocitrate	4.402 (2.249–7.266)	<0.0001

Abbreviations: BD, bipolar disorder; CI, confidence interval; CSF, cerebrospinal fluid.

Logistic function, *P*={1 + exp (5.3463−1.3967*X*)}, where *P* is probability of being statistically discriminated as BD, and *X* is isocitrate.

**Table 3A:** Expression of *ACO* and *IDH* genes in the post-mortem brain samples

*Genes*	*Controls (*n=*34)*	*BD (*n=*35)*	P-*value (unpaired* t*-test)*	P-*value (false discovery rate)*
*Gene expression of ACO and IDH genes in the dorsolateral prefrontal cortex*				
* ACO1*	1.083±0.205	1.171±0.315	0.178	0.414
* ACO2*	0.922±0.149	0.897±0.142	0.487	0.568
* IDH1*	1.208±0.337	1.233±0.333	0.755	0.755
* IDH2*	1.316±0.544	1.186±0.437	0.279	0.488
*** IDH3A***	**1.466±0.832**	**1.017±0.416**	0.007**	0.026*
*** IDH3B***	**1.198±0.420**	**0.957±0.287**	0.007**	0.026*
* IDH3G*	1.084±0.174	1.049±0.183	0.422	0.568

Abbreviations: ANOVA, analysis of variance; BD, bipolar disorder; MDD, major depressive disorder.

The data are the mean±s.d.

*P<0.05 (false discovery rate).

**P<0.01 (unpaired *t*-test). The bold is statistically significant.

**Table 3B:** Expression of IDH3A and IDH3B proteins in the post-mortem brain samples

*Proteins*	*Controls (*n=*15)*	*BD (*n=*15)*	*MDD (*n=*15)*	*Schizophrenia (*n=*15)*	*One-way ANOVA*
*Protein expression of IDH3A and IDH3B in the cerebellum and parietal cortex*					
* Cerebellum*					
** IDH3A**	**1.227±0.543**	0.804±0.390*	0.746±0.356**	0.660±0.303**	**F(3, 56)=5.74,** ***P=*****0.002**
IDH3B	1.020±0.883	0.839±0.373	0.605±0.243	0.610±0.255	F(3, 56)=2.30, *P*=0.087
*Parietal cortex*					
IDH3A	0.951±0.527	0.672±0.310	0.648±0.435	0.564±0.307	F(3, 56)=2.58, *P*=0.063
IDH3B	0.738±0.311	0.621±0.330	0.760±0.560	0.602±0.300	F(3, 56)=0.63, *P*=0.597

Abbreviations: ANOVA, analysis of variance; BD, bipolar disorder; MDD, major depressive disorder.

The data are the mean±s.d.

*P<0.05, **P<0.01 (vs control, Dunnett's test). The bold is statistically significant.
