# Supplementary material for: Cerebrospinal fluid metabolomics identifies a key role of isocitrate dehydrogenase in bipolar disorder: evidence in support of mitochondrial dysfunction hypothesis
Source: Mol Psychiatry. 2016 Jan 19;21(11):1504–10. doi: 10.1038/mp.2015.217 (PMC5078854; doi:10.1038/mp.2015.217)
Supplement: Supplementary Information [file mp2015217x12.doc]

**Supplemental Methods**

**Metabolomic analyses of human samples**

The volume (50 µL) of CSF sample was added to 450 µL methanol containing internal standards, and mixed. Then, 450 µL chloroform and 200 µL Milli-Q water was added to the mixture. After mixture, the mixture was centrifuged at 2,300 ×*g* and 4ºC for 5 min. Subsequently, 800 µL of upper aqueous layer was centrifugally filtered through a Millipore 5-kDa cutoff filter at 9,100 ×*g* and 4ºC for 120 min to remove proteins. The filtrate was centrifugally concentrated and re-suspended in 25 µL of Milli-Q water for analysis.

Cationic compounds were measured in the positive mode of CE-TOFMS (Agilent CE-TOFMS system Machine No. 3, Fused silica capillary, i.d. 50 µM x 80 cm), and anionic compounds were measured in the positive and negative modes of CE-MS/MS (Agilent CE system and Agilent 6400 TripleQuad LC/MS Machine No. QqQ01, Fused silica capillary, i.d. 50 µM x 80 cm), as reported previously (1-3). Peaks detected by CE-TOFMS and CE-MS/MS were extracted using automatic integration software (MasterHands, Keio University, Tsuruoka, Japan) (4) and MassHunter Quantitative Analysis B.04.00, Agilent Technologies, Santa Clara, CA, USA) in order to obtain peak information including *m/z*, migration time (MT), and peak area. The peaks were annotated with putative metabolites from the HMT metabolite database based on their MTs in CE and *m*/*z* values determined by TOFMS. The tolerance range for the peak annotation was configured at ± 0.5 min for MT and ± 10 ppm for *m/z*. In addition, concentrations of metabolites were calculated by normalizing the peak area of each metabolite with respect to the area of the internal standard and by using standard curves, which were obtained by three-point calibrations. In this study, 116 major metabolic substances from several pathways (glycolytic system, pentose phosphate pathway, citric acid cycle, urea cycle, polyamine-creatine metabolism pathway, purine metabolism pathway, glutathione metabolism pathway, nicotinamide metabolism pathway, choline metabolism pathway and diverse amino acid metabolism pathway) were selected for metabolomic analyses (**Table S1 and Table S2**).

**Expression of *IDH* and *ACO* genes in the dorsolateral prefrontal cortex**

Diagnoses were made in accordance with DSM-IV criteria. There were no significant differences in RNA Integrity Number (RIN) values (**Table S3**), which is a critical cofounding factor for mRNA expression analysis, between the BD and control groups. All BD patients had previously received therapeutic drugs to treat their disease. Real-time quantitative RT-PCR analysis was conducted using an ABI7900HT Fast Real-Time PCR System (Applied Biosystems, Foster City, CA) in both human and rat samples. TaqMan probes and primers for the seven genes (*ACO1, ACO2, IDH1, IDH2, IDH3A, IDH3B, IDH3G*) and *GAPDH* (an internal control) (**Table S11**) were TaqMan® Gene Expression Assays products (Applied Biosystems). All real-time quantitative RT-PCR reactions were performed in triplicate, based on the standard curve method (5).

**Protein expression of IDH3A and IDH3B in the cerebellum and parietal cortex of BP patients and controls**

Diagnoses were made in accordance with DSM-IV criteria. There were no significant demographic differences between the bipolar disorder and control groups, in terms of age, PMI, sample pH, and brain weight (**Table S4**). The tissues were homogenized in RIPA lysis buffer containing 150 mM NaCl, 25 mM Tris–HCl pH 7.6%, NonidetP-40, 1% sodium deoxycholate, 1% SDS and protease inhibitor. The homogenate was sonicated in a cold pack. Lysates were centrifuged and the supernatants were collected and stored at -80°C until use. The concentration of protein was determined using the BCA protein assay Reagent (Thermo Fischer Scientific). Forty micrograms of protein were electrophoresed on Mini-PROTEAN® TGX Stain-Free™ Precast Gels (Bio-Rad Laboratories, Inc). Proteins were electro transferred onto PVDF membranes. The nonspecific binding of immunoproteins was blocked with 5% non-fat dry powdered milk dissolved in Tris-buffered saline Tween-20 (TBS-T) for 1h at room temperature (RT). After blocking, the membranes were incubated with primary antibodies for 1h at RT. Antibodies were dissolved in TBS-T containing 5% non-fat powdered milk. The primary antibodies were: anti-IDH3A: 1:1000, #ab109686 (Abcam); anti-IDH3B: 1:500, #SAB2501848 (Sigma -Aldrich). The membranes were rinsed in TBS-T followed by 1h incubation with HRP-conjugated secondary antibody at RT. After incubation, the membranes were repeatedly washed in TBS-T and incubated with an enhanced chemiluminescence reagent with ECL Prime Western Blotting Detection Reagent (GE Healthcare). The protein bands were visualized with ImageQuant LAS3000mini (GE Healthcare). The bands were quantified by Multi Gauge Ver3.0 (Fujifilm), and standardized to α-tubulin (1:1000, #2144 Cell Signaling). With this software the optical density of the protein bands was measured. Results were expressed in relative density units. All data were performed in duplicate.

**Geneic analyses of *IDH* and *ACO* genes in BD patients and controls**

BD subjects were collected through three channels. The BD cases (n=1416) were identified using the Swedish National Quality Assurance Registry for Bipolar Disorder (BipoläR) (6, 7). Additional subjects were recruited from the Bipolar outpatient clinic at the Northern Stockholm Psychiatry Clinic, Sweden, following physician’s referral for BD (n=315). The diagnostic instrument used was a Swedish adaptation of the Affective Disorder Evaluation (8), which includes the affective module of the SCID. A further 576 BD cases were recruited from the Stockholm County catchment area, and diagnoses were made according to the DSM-IV criteria. Control subjects, also selected through registers, were group-matched by age, sex and county of residence, and must not have been hospitalized with a psychiatric diagnosis. All subjects were at least 18 years old and gave written informed consent to participate. The study was also approved by the Ethical Committee at Karolinska Institutet.

Blood samples were obtained and DNA extracted from whole blood using standard methods at Karolinska Institutet. Samples were genotyped using one of two arrays: Illumina OmniExpress for Sample 1 (Illumina, Inc. San Diego, CA, USA) or Affymetrix 6.0 for Sample 2 (Affymetrix, Santa Clara, CA, USA). All genotyping was conducted at the Broad Institute of Harvard and MIT, and genotypes were called using the Birdsuite algorithm (9). The quality control exclusionary measures for subjects were: genotype call rates <95%; ancestry outliers via multidimensional scaling; a randomly selected member of any pair of subjects with high relatedness (pi-hat>.20); and suspected sample error or contamination. SNPs were excluded for marked departure from Hardy-Weinberg equilibrium (P < 1 x 10^-6^), low minor allele frequencies (< 1%), and non-random genotyping failure, inferred from the flanking haplotype background using the PLINK ‘mishap’ test (P < 1 x 10^-10^). Plate-based associations of P < 1 x 10^-6^ were taken as evidence of non-random plate failure, based on a comparison of allele frequency of each plate to all others and were removed on a plate-by-plate basis. Following quality control steps, Sample 1 consisted of 1415 cases and 1271 controls, and Sample 2 contained 836 cases and 2093 controls.

We imputed our genotypes against autosomal genotype data from HapMap3 (10) using BEAGLE (11). All association analyses were conducted using logistic regression in PLINK (12). Multidimensional scaling was performed on the entire data set, and each collection sample was analyzed separately using the first four multidimensional scaling components as covariates, to control for population substructure.

Epistasis tests were also conducted in PLINK separately by sample using the directly genotyped markers. Interactions between the markers within the sets of *IDH* and *ACO* genes were tested, yielding 272 tests in sample 1 and 152 tests in sample 2.

**Metabolomics of rat CSF samples**

Male Sprague-Dawley rats (Japan SLC Inc., Shizuoka, Japan) aged 6 weeks old were used in this study. All rats were housed in groups of three per cage in a room maintained at 23 °C ± 2 °C and 60 ± 10 % humidity with a 12/12 h light/dark cycle (lights on at 7:00 a.m.). The rats were given free access to food and water. Animal care and use were conducted in accordance with the Institutional Guidelines for Animal Care and Use of Otsuka Pharmaceutical Co., Ltd. (Tokushima, Japan). Animals were divided into three groups (n=6), with the treatment group receiving lithium carbonate (Li; 600 mg/L, Wako Pure Chemical Industries, Ltd., Osaka, Japan) or valproic acid sodium salt (VPA: 4 g/L, Sigma-Aldrich Co., Tokyo, Japan) via drinking water, and the control group receiving vehicle (water) for 4 weeks (day 1- day 28). The drinking water was replaced twice a week. The doses of Li and VPA were selected based on the doses that were previously reported (13, 14). All animals were decapitated on day 30 after CSF sampling, and brain regions (prefrontal cortex, and hippocampus) were dissected rapidly on ice. Metabolomic analyses of rat CSF samples were performed as described above.

**Expression of *Idh* and *Aco* genes of rat brain samples**

Prefrontal cortex, and hippocampus were dissected rapidly on ice and stored in RNA later (Applied Biosystems) at 4 °C until used for RNA isolation, cDNA synthesis and polymerase chain reaction (PCR) amplification. The total RNA was extracted using the RNeasy Lipid Tissue Mini Kit (QIAGEN) following the instructions of manufacturer. RNA yield and integrity was assessed using Nanodrop (NanoDrop Technologies). Reverse transcription of total RNA was done using the SuperScript VILO cDNA Synthesis Kit (Invitrogen). In the quantitative PCR, the cDNA was amplified using commercial TaqMan assays (Applied Biosystems) for rat *Idh3A* (Rn00586270_m1), *Idh3B* (Rn00504589_g1), *Aco1* (Rn00569045_m1), *Aco2* (Rn00577876_m1) and *Gapdh* (Rn01775763_g1) with an ABI 7500 Fast Real-Time PCR system (Applied Biosystems). Reactions were performed in triplicates. All reactions were normalized to *Gapdh* and presented as relative expression changes compared to control.

**References**

1. Soga T, Heiger DN. Amino acid analysis by capillary electrophoresis electrospray ionization mass spectrometry. *Anal Chem* 2000; **72(6):** 1236-1241.
2. Soga T, Ueno Y, Naraoka H, Ohashi Y, Tomita M, Nishioka T. Simultaneous determination of anionic intermediates for Bacillus subtilis metabolic pathways by capillary electrophoresis electrospray ionization mass spectrometry. *Anal Chem* 2002; **74(10):** 2233-2239.
3. Soga T, Ohashi Y, Ueno Y, Naraoka H, Tomita M, Nishioka T. Quantitative metabolome analysis using capillary electrophoresis mass spectrometry. *J Proteome Res* 2003; **2(5):** 488-494.
4. Sugimoto M, Wong DT, Hirayama A, Soga T, Tomita M. Capillary electrophoresis mass spectrometry-based saliva metabolomics identified oral, breast and pancreatic cancer-specific profiles. *Metabolomics* 2010; **6(1):** 78-95.
5. Yamada K, Iwayama Y, Toyota T, Ohnishi T, Ohba H, Maekawa M, *et al.* Association study of the KCNJ3 gene as a susceptibility candidate for schizophrenia in the Chinese population. *Hum Genet* 2012; **131**(3): 443-451.
6. Karanti A, Bobeck C, Osterman M, Kardell M, Tidemalm D, Runeson B, *et al.* Gender differences in the treatment of patients with bipolar disorder: a study of 7354 patients. *J Affect Disord* 2015; **174:** 303-309.
7. Sellgren C, Landen M, Lichtenstein P, Hultman CM, Langstrom N. Validity of bipolar disorder hospital discharge diagnoses: file review and multiple register linkage in Sweden. *Acta Psychiatr Scand* 2011; **124(6):** 447-453.
8. Ryden E, Thase ME, Straht D, Aberg-Wistedt A, Bejerot S, Landen M. A history of childhood attention-deficit hyperactivity disorder (ADHD) impacts clinical outcome in adult bipolar patients regardless of current ADHD. *Acta Psychiatrica Scand* 2009; **120(3):** 239-246.
9. Korn JM, Kuruvilla FG, McCarroll SA, Wysoker A, Nemesh J, Cawley S, *et al.* Integrated genotype calling and association analysis of SNPs, common copy number polymorphisms and rare CNVs. *Nat Genet* 2008; **40(10):** 1253-1260.
10. Altshuler DM, Gibbs RA, Peltonen L, Altshuler DM, Gibbs RA, Peltonen L, *et al.* Integrating common and rare genetic variation in diverse human populations. *Nature* 2010; **467(7311):** 52-58.
11. Browning SR, Browning BL. Rapid and accurate haplotype phasing and missing-data inference for whole-genome association studies by use of localized haplotype clustering. *Am J Hum Genet* 2007; **81(5):** 1084-1097.
12. Purcell S, Neale B, Todd-Brown K, Thomas L, Ferreira MA, Bender D, *et al.* PLINK: a tool set for whole-genome association and population-based linkage analyses. *Am J Hum Genet* 2007; **81(3):** 559-575.
13. Talab SS, Emami H, Elmi A, Nezami BG, Assa S, Deroee AF, *et al.* Chronic lithium treatment protects the rat kidney against ischemia/reperfusion injury: the role of nitric oxide and cyclooxygenase pathways. *Eur J Pharmacol* 2010; **647(1-3):** 171-177.
14. Niles LP, Sathiyapalan A, Bahna S, Kang NH, Pan Y. Valproic acid up-regulates melatonin MT1 and MT2 receptors and neurotrophic factors CDNF and MANF in the rat brain. *Int J Neuropsychopharmacol* 2012; **15(9):** 1343-1350.
